# High Socioeconomic Status and Key Risk Factors of Overweight and Obesity among Rural-to-Urban Migrant 7-10y-old Children in Shanghai, China

**Published:** 2017-03

**Authors:** Jinkui LU, Xiaojian YIN, Yuqiang DENG, Liu JI

**Affiliations:** 1. Key Laboratory of Adolescent Health Assessment and Exercise Intervention of Ministry of Education, East China Normal University, Shanghai 200241, PR China; 2. School of Physical Education & Health Care, East China Normal University, Shanghai 200241, PR China; 3. School of Sport and Science, Nantong University, Nantong, 226019, PR China

## Dear Editor-in-Chief

Obesity has become a global epidemic problems, especially childhood obesity is a key public health issue in both the developed and developing countries (1), “because antecedents of adult disease (hypertension, hyperlipidemia, and so on) occur with increased frequencies in obese children and adolescents” (2, 3). In china, migrant children have prevalence of overweight and obesity and it has an upward tendency (4, 5). To our knowledge, a large number of studies on children and adolescents have examined that the relationship between obesity and socioeconomic status (SES) and lifestyle behaviors’ factors (6, 7), but few research revealed relationship between them among rural-to-urban migrant children.

This study aimed to examine the relationship between overweight and obesity and socioeconomic status as well as lifestyle behaviors’ factors among rural-to-urban migrant children in Shanghai, China. A sample of 1705 aged 7–10 yr old rural-to-urban migrant students was selected randomly from 12 public schools and 162 classes in 2013.

Informed consent was obtained from the parents of all subjects. The study was approved by the Ethics Committee of School of Physical Education and Health, East China Normal University.

Physical measurement and questionnaire investigation was conducted. SPSS 17.0 (version 17, SPSS, Inc., Chicago, IL, USA) was used for the analyses. Data were expressed as mean for the normal distributions or median if normal distribution was rejected. Chi-square or Fisher’s exact test was used to compare the categorical variables. Student’s t test or Wilcoxon two-sample test was applied to compare continuous variables between the two groups. Logistic Regression model was used in risk factor analyses.

No gender difference towards the BMI was found and weight status of the participants was divided into four levels (Underweight, Normal weight; overweight and obesity), significant difference of duration of living in Shanghai was found among four groups both for boys and girls, highest proportion of boys who lived in Shanghai for a short and medium duration were found to be underweight while more than half of the boys (51.3%) lived for a long duration showed to be obese in this study. The same trend was found in girls (52.4%). Moreover, scatter diagram between body fat percentage and duration of living in Shanghai confirmed the previous founding. Different education level of father and mother distributed differently among the four weight status in all the participants. The highest proportion (58.8%) of obese sons had manual labor fathers, while it was contrary for non-manual labor fathers. But highest proportion of overweight daughters and obese daughters corresponded to manual and non-manual labor fathers, 62.3%, 52.3% respectively. Most of the obese sons corresponded to non-manual labor mothers, most of the obese daughters corresponded to non-manual labor mothers.

Most of the obese boys were from high income families. As for girls, most of the obese ones came from medium income family; most of the overweight girls came from high income families. Inactive physical exercise parents corresponded to obese children. While no difference for the status of smoking and drinking status of the parents was found for all the weight status children. Logistic regression analyses results confirmed that the factors as duration of living in Shanghai, parental educational level, family income and parental status of physical exercises may affect the status of the weight status of the children.

Therefore, long duration of living in Shanghai, high socioeconomic factors are the key risk of overweight and obesity among rural-to-urban migrant 7–9 yr-old children.
